# Antioxidant Compound, Oxyresveratrol, Inhibits APP Production through the AMPK/ULK1/mTOR-Mediated Autophagy Pathway in Mouse Cortical Astrocytes

**DOI:** 10.3390/antiox10030408

**Published:** 2021-03-08

**Authors:** Md. Ataur Rahman, Yoonjeong Cho, Ghilsoo Nam, Hyewhon Rhim

**Affiliations:** 1 Center for Neuroscience, Brain Science Institute, Korea Institute of Science and Technology (KIST), 5 Hwarang-ro 14-gil, Seongbuk-gu, Seoul 02792, Korea; rahman@kist.re.kr (M.A.R.); h14009@kist.re.kr (Y.C.); 2 Center for Neuro-Medicine, Korea Institute of Science and Technology (KIST), Seoul 02792, Korea; gsnam@kist.re.kr; 3 Division of Bio-Medical Science and Technology, KIST School, Korea University of Science and Technology (UST), Seoul 02792, Korea

**Keywords:** oxyresveratrol, amyloid precursor protein (APP), AMPK-ULK1, mTOR, LC3 puncta, autophagy

## Abstract

Oxyresveratrol (OxyR), a well-known polyphenolic phytoalexin, possesses a wide range of pharmacological and biological properties, comprising antioxidant, anti-inflammatory, free radical scavenging, anti-cancer, and neuroprotective activities. Autophagy is a cellular self-degradation system that removes aggregated or misfolded intracellular components via the autophagosome-lysosomal pathway. Astrocyte accumulation is one of the earliest neuropathological changes in Alzheimer’s disease (AD), and amyloid precursor protein (APP) is the hallmark of AD. OxyR could affect APP modulation via the autophagy pathway. Here, we have reported that OxyR promotes autophagy signaling and attenuates APP production in primary cortical astrocytes based on immunofluorescence and immunoblotting assay results. Co-treatment with the late-stage autophagy inhibitor chloroquine (CQ) and OxyR caused significantly higher microtubule-associated protein light chain 3 (LC3)-II protein levels and LC3 puncta counts, demonstrating that OxyR stimulated autophagic flux. We also found that OxyR significantly reduced the levels of the autophagy substrate p62/SQSTM1, and p62 levels were significantly augmented by co-treatment with OxyR and CQ, because of the impaired deficiency of p62 in autolysosome. Likewise, pretreatment with the autophagy inhibitor, 3-methyladenine (3-MA), resulted in significantly fewer OxyR-induced LC3 puncta and lower LC3-II expression, suggesting that OxyR-mediated autophagy was dependent on the class III PI3-kinase pathway. In contrast, OxyR caused significantly lower LC3-II protein expression when pretreated with compound C, an AMP-activated protein kinase (AMPK) inhibitor, indicating that AMPK signaling regulated the OxyR-induced autophagic pathway. Additionally, co-treatment with OxyR with rapamycin intended to inhibit the mammalian target of rapamycin (mTOR) caused significantly lower levels of phospho-S6 ribosomal protein (pS6) and higher LC3-II expression, implying that OxyR-mediated autophagy was dependent on the mTOR pathway. Conversely, OxyR treatment significantly upregulated unc-51-like autophagy activating kinase 1 (ULK1) expression, and ULK1 small interfering RNAs (siRNA) caused significantly lower OxyR-induced LC3 puncta counts and LC3-II expression, indicating that ULK1 was essential for initiating OxyR-induced autophagy. However, we found that OxyR treatment astrocytes significantly increased the expression of lysosome-associated membrane protein 1 (LAMP1). Finally, we established a stress-induced APP production model using corticosterone (CORT) in cortical astrocytes, which produced significantly more APP than the equivalent using dexamethasone (DEX). In our experiment we found that CORT-induced APP production was significantly attenuated by OxyR through the autophagy pathway. Therefore, our study reveals that OxyR regulates AMPK/ULK1/mTOR-dependent autophagy induction and APP reduction in mouse cortical astrocytes.

## 1. Introduction

The naturally occurring polyphenolic compound, oxyresveratrol (OxyR, trans-2,3′,4,5′-tetrahydroxystilbene), is known as a strong free radical scavenger [1], and is readily available from the mulberry plant, *Morus alba* L. [2]. OxyR has been demonstrated to effectively scavenge H_2_O_2_, NO, and the artificial free radical DPPH (2,2-diphenyl-l-picrylhydrazyl) in microglial cells [3]. OxyR has been shown to inhibit neuronal apoptotic cell death in neuroblastoma [4] and cerebral ischemia models [1]. Moreover, OxyR has been shown to cross the blood-brain barrier and to exert direct protective effects in the brain, rendering it an excellent drug candidate for the treatment of neurodegenerative disorders [5]. It has been reported that 10 µM of OxyR inhibits amyloid β (Aβ) expression and prevents neuronal cell death via the elevation of cytosolic calcium (Ca^2+^) concentration [6]. OxyR has also been found to repress glutamate release and reduce the generation of reactive oxygen species (ROS) into the medium, which may be related to its neuroprotective activities [6]. OxyR has been found to decrease amyloid β (Aβ) production through the inhibition of β-secretase [7]. However, no in vivo study has examined the neuroprotective effects of OxyR in Alzheimer’s disease (AD); additional studies are therefore warranted, aimed at exploration of the neuroprotective target of OxyR. The aim of this study was to examine whether OxyR conferred protection against corticosterone (CORT)-induced amyloid precursor protein (APP) production through the autophagy pathway in cortical astrocytes.

APP is sequentially cleaved by the action of β-secretase and γ-secretase, producing Aβ peptide that forms plaques, which are the hallmark of AD pathogenesis [8]. Even though the majority of studies have focused on neuronal Aβ levels in AD [9], the presence of reactive astrocytes has been shown to promote more efficient Aβ deposition during the early stages of AD [10]. However, the origin and consequence of this functional role have not been fully clarified thus far. Astrocytes are star-shaped glial cells that play essential roles in neuronal development, survival, and biochemical support and repair, by enabling the removal of dead neurons and elimination of pathogens, as well as maintenance of synaptic connection and homeostasis [11]. It has also been reported that astrocytes are capable of expressing APP as well as β-site APP-cleaving enzyme (BACE1) [12]. Although low levels of astrocytic Aβ accumulation may contribute to the Aβ burden in AD, astrocytes engulf synapses, dead cells, and Aβ aggregates during AD pathogenesis [13,14]. Additionally, activated astrocytes are reported to enhance Aβ generation via BACE1 [15]. Therefore, reactive astrocytes may constitute an essential cellular source of Aβ peptides in the AD brain [10]. Furthermore, astrocytes are proteolytic, migratory, and phagocytic in nature [12]. All these cellular functions are neuroprotective because they play an important role in degradation and clearance of Aβ during AD pathogenesis [16]. Furthermore, it has been found that glucocorticoids (GCs) may contribute to the maintenance and development of AD when basal cortisol levels are elevated in AD patients [17]. Particularly, it has been mentioned that GC administered to normal and middle-aged mice stimulates APP expression in astrocytes [17]. Thus, we hypothesized that GC-induced APP accumulation in astrocytes might be diminished by autophagy, although the formation and regulation of APP by GC stress is not fully understood.

Autophagy is a cellular process that disintegrates unwanted or aggregated dysfunctional cellular components by lysosomal fusion and is essential to maintaining cellular function as well as homeostasis [18]. Generally, autophagy is initiated via membrane nucleation, which results in phagophore formation. AMP-activated protein kinase (AMPK) has been reported to regulate autophagy initiation [19]. Importantly, the mammalian target of rapamycin (mTOR), regulated by nutrients and growth factors, has been found to initiate autophagy and is required for neuronal function [20]. AMPK has been implicated in the stimulation of autophagy through the inactivation of mTOR [21]. However, several studies have indicated that the unc-51-like autophagy activating kinase 1 (ULK1) complex (ULK1/Atg13/Atg101/FIP200), a serine/threonine-protein kinase, regulates autophagy initiation [22,23]. During the autophagy process, the microtubule-associated protein light chain 3 (LC3) is involved as follows: pro-LC3 is converted to LC3-I, which binds to phosphatidylethanolamine to form LC3-II, which ultimately stimulates phagophore elongation in addition to autophagosome formation [24]. Subsequently, autolysosomes, which are fusions of autophagosomes with lysosomes, are degraded and recycled via autophagy. Therefore, monitoring LC3-II formation and degradation serves as a reliable method for monitoring the overall autophagy process [25,26]. Recently, autophagy has been recognized as being closely associated with the development of neurodegenerative disorders [27,28]; however, few studies have been conducted regarding APP reduction in association with autophagy modulation in astrocytes. In the present study, we focused on examining the effect of OxyR administration on astrocyte autophagy signaling. Our results demonstrated that OxyR considerably increased autophagy via AMPK/ULK1/mTOR-dependent pathways in primary cultured astrocytes. In contrast, OxyR treatment markedly activated lysosomal protein LAMP1 and autophagic flux formation. Furthermore, OxyR decreased CORT-induced APP expression via the autophagy pathway in mouse cortical astrocytes. Therefore, our findings revealed a novel molecular mechanism, showing that CORT-induced APP production was reduced by the OxyR-mediated autophagy pathway, which might contribute to our understanding of AD pathogenesis.

## 2. Materials and Methods

### 2.1. Reagents

Oxyresveratrol, 3-methyladenine (3-MA), rapamycin, chloroquine diphosphate salt (CQ), and dexamethasone (DEX) were purchased from Sigma-Aldrich (St. Louis, MO, USA). Anti-LC3 (D3U4C) XP Rabbit mAb (Alexa Fluor 488 Conjugate), anti-SQSTM1/p62, anti-phospho-S6, anti-total S6, anti-LAMP1, APP, and anti-LC3 antibodies were purchased from Cell Signaling Technology (Danvers, MA, USA). Anti-MAP2 (microtubule-associated protein 2) antibody (ab5392), goat anti-chicken IgY H&L (Alexa Fluor 647) (ab150171), and corticosterone (ab14597) were obtained from Abcam (Cambridge, UK). Anti-rabbit HRP-linked secondary antibody (#7074P2) and goat anti-mouse HRP-linked secondary antibody (#62-6520) were purchased from Sigma-Aldrich and Invitrogen (Carlsbad, CA, USA), respectively. ULK1 siRNA (m): (sc-44849) was purchased from Santa Cruz Biotechnology, Inc. (Santa Cruz, CA, USA). OxyR, CQ, rapamycin, and compound C were dissolved in dimethyl sulfoxide and 3-MA was dissolved in double-distilled water.

### 2.2. Cortical Astrocyte Culture

Cortical astrocytes were cultured using our previously established methods [11,25]. Briefly, 1-day-old (P1) ICR mice (Orient Bio Inc., Seoul, Korea) were used as the astrocyte source. The brain was carefully isolated and separated in Hank’s buffered salt solution containing streptomycin and penicillin. After careful removal of the cerebral hemispheres, 0.1% trypsin-0.05% Ethylenediaminetetraacetic acid (EDTA) was added and the specimens were incubated for 25 min at 37 °C. The tissues were inverted at 5-min intervals. After 25 min of incubation, the brain tissues were centrifuged at 1000× *g* rpm for 3 min, the supernatant was removed, and then detrypsinization in Dulbecco’s modified Eagle medium (DMEM) was performed. The cells were then centrifuged at 1000× *g* rpm for 3 min and subjected to washing steps using 1 mL fresh DMEM. The tissues were dissociated by Pasteur pipette size adjustment and centrifuged at 1000× *g* rpm for 3 min. After the supernatants were discarded, the cells were seeded in a 100-mm culture dish in DMEM containing fetal bovine serum (FBS) (10%) and horse serum (10%) and were incubated for growth at 37 °C in a 5% CO_2_ atmosphere. After 5 d, the culture dishes were shaken manually to remove loosely attached neuronal cells and fresh medium was added. These astrocytes were used for conducting the subsequent experiments.

### 2.3. Cortical Neuron Cultures

Cortical neurons were prepared using a modified version of the technique reported by Cho et al. [29]. Briefly, cells were collected from 18-day-old Sprague-Dawley rat fetuses. The isolated cortex was incubated in 0.25% pre-warmed trypsin solution at 37 °C for 25 min, and the cells were inverted at 5-min intervals. After centrifugation, the cells were mechanically separated using fire-polished Pasteur pipettes, and then plated in 12-well and 6-well dishes coated with poly-d-lysine. Cells were cultured in neurobasal medium (#21103-049) containing 2% B-27 supplement, glucose 4500 mg/L, 5% FBS, 2 mM glutamine, 100 U/mL penicillin, and 100 μg/mL streptomycin, in a humidified atmosphere of 95% air and 5% CO_2_ at 37 °C. Subsequently, the media were changed to the same composition without FBS twice a week and the cells were treated with fluorodeoxyuridine (10 μM) after 7 d in vitro. All experiments on cortical neurons were performed after 14 d of in vitro culture.

### 2.4. Immunocytochemical Analysis

After treatment, the cells were subjected to washing steps using a phosphate-buffered saline (PBS) solution and fixed with 100% methanol at −20 °C for 15–20 min. After fixation, cells were washed three times with PBS. A block solution was added and the cells were incubated for 1 h with 5% normal goat serum containing 0.3% Triton X-100 in PBS. After subjection to blocking, cells were incubated with anti-GFAP (glial fibrillary acidic protein) conjugate with Alexa Fluor 555 (1:50) and Alexa Fluor 488 conjugate anti-LC3-II (1:50) for astrocytes, as well as the neuronal marker MAP2 (1:2000) and goat anti-chicken Alexa Fluor 647 for neuronal cells in 1% bovine serum albumin (BSA), and 0.3% Triton X100 PBS overnight at 4 °C. The next day, the nuclear marker 4′,6-diamidino-2-phenylindole (DAPI) (1 µg/mL) in PBS was added to the cells and incubation was performed for 10 to 15 min. After performing mounting and drying procedures, LC3-II puncta were imaged by confocal microscopy using the Leica Application Suite X (LAS X) microscope (Leica Microsystems, Wetzlar, Germany). Further details of the methods used can be found in studies authored by Rahman et al. [11,25].

### 2.5. ULK1 Small Interfering RNA (siRNA) Transfection

Transient transfection was performed using ULK1 siRNAs and Lipofectamine2000 according to the manufacturer’s instructions (Invitrogen, Carlsbad, CA, USA). Specifically, astrocytes were cultured in a 6-well plate with 2 mL of DMEM without antibiotics. Lipofectamine2000 was mixed and diluted with normal DMEM and kept at room temperature for 5 min. A final concentration of 50 nM siRNA oligomers was added to the above-mentioned medium for 20 min. A complex mixture of siRNA and Lipofectamine2000 was added to the astrocyte-containing medium and incubation was conducted for 48 h at 37 °C. Experiments using scrambled siRNA with a final concentration of 50 nM were considered as controls. After successful transfection, astrocytes were used for conducting immunoblotting and immunocytochemistry studies.

### 2.6. Immunoblotting

To conduct immunoblotting, astrocytes and neurons were cultured in a 6-well dish. When the cells exhibited maturity, treatment with the drug was performed. After 24 h, the cells were harvested using a radioimmunoprecipitation assay buffer (Elpis-Biotech. Inc., Daejeon, Korea). The collected cell lysates were kept on ice for 30 min. Lysates were centrifuged at 14,500× *g* rpm for 10 min at 4 °C. The upper layer of proteins was separated from the tube. Protein quantification was performed using Bradford (Coomassie) protein assay kits (Gendepot, Katy, TX, USA). Reducing gels (8–15% depending on the protein size) were prepared. Equivalent quantities of protein samples were loaded into each well and protein were separated by sodium dodecyl sulfate-poly acrylamide gel electrophoresis (SDS-PAGE). After separation, the proteins were transferred onto a polyvinylidene fluoride (PVDF) membrane. Subsequently, the membrane was blocked using 5% skim milk or BSA for 1 h at room temperature. After completion of blocking, each membrane was subjected to washing steps with phosphate-buffered saline with 0.1% Tween 20 (PBST) and incubated with a specific primary antibody at 4 °C overnight. The membrane was then rinsed three times with PBST. Next, 5% skim milk or BSA was used to dissolve the secondary antibody conjugated with horseradish peroxide, and the membrane was incubated for a minimum of 2 h at room temperature. Finally, the membrane was rinsed three times with PBST, and the bands were detected with enhanced chemiluminescence kits using the AlphaEase program (Alpha Innotech, San Leandro, CA, USA). In all experiments, the band intensities were quantified using ImageJ software.

### 2.7. Autophagic Flux Evaluation

Puncta were counted and their formation analyzed using immunocytochemistry images acquired under a confocal microscope, as per methods described in a previous study [11]. Briefly, a minimum of five cells were subjected to puncta evaluation from each image per condition, and the average counts were plotted as bar graphs. Measurements of autophagic flux in vitro were performed using the autophagosomal or lysosomal fusion inhibitor CQ before the cells were harvested or fixed, and then probing was performed using the anti-LC3-II conjugate Alexa Fluor 488.

### 2.8. Statistical Analysis

Two-group comparisons were performed using unpaired Student’s *t*-tests. Multiple comparisons were performed using one-way analysis of variance (ANOVA) followed by Tukey’s multiple comparisons post hoc test. GraphPad Prism 7 software (GraphPad Software Inc., La Jolla, CA, USA) was used for all data analyses. Data are presented as mean ± SEM. Results were considered statistically significant at * *p* < 0.05, ** *p* < 0.01, *** *p* < 0.001, and **** *p* < 0.0001.

## 3. Results

### 3.1. OxyR Treatment Activates Autophagic Flux in Mouse Cortical Astrocytes and Rat Cortical Neurons

**Figure 1:**
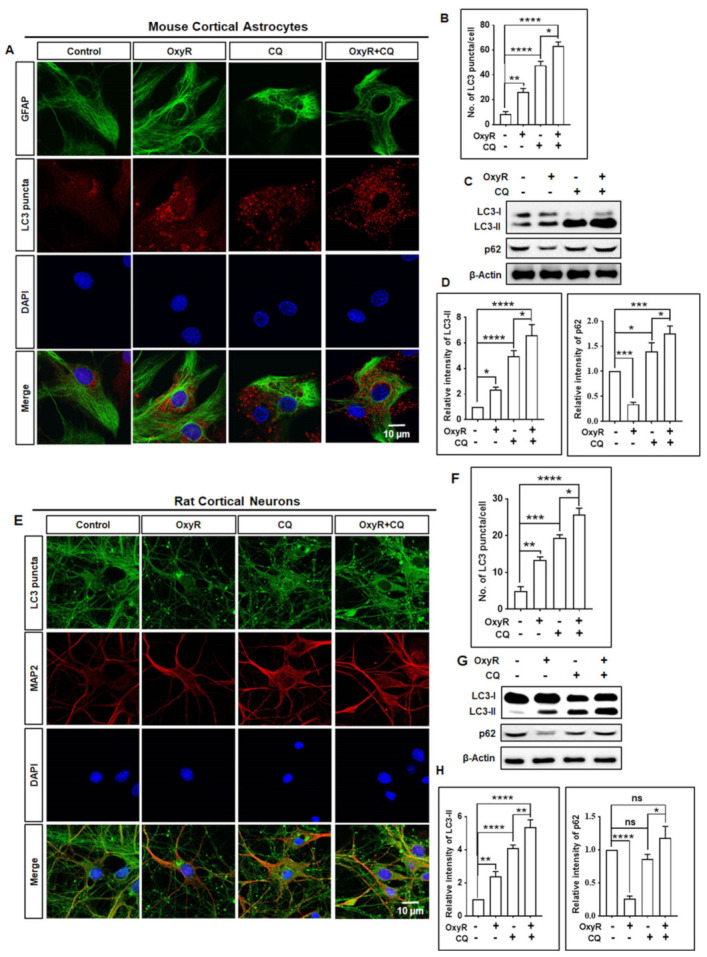
OxyR treatment induced autophagic flux in cortical astrocytes and neurons. (**A**,**E**) Before harvest, cells were treated with 10 µM CQ for 2 h. Images were acquired using a confocal microscope. (**B**,**F**) Numbers of LC3 puncta were counted and analyzed using one-way ANOVA (*n* = 3). (**C**,**G**) Before harvest, CQ treatment for 2 h was performed and representative LC3 and p62 levels were determined by Western blotting. (**D**,**H**) Statistically significant differences were determined by one-way ANOVA (*n* = 3). Data are presented as mean ± SEM. * *p* < 0.05, ** *p* < 0.01, *** *p* < 0.001, **** *p* < 0.0001, ns: non-significant. OxyR, oxyresveratrol; CQ, chloroquine; GFAP, glial fibrillary acidic protein; LC3, microtubule-associated protein light chain 3; DAPI, the nuclear marker 4′,6-diamidino-2-phenylindole.

To analyze OxyR-mediated autophagic flux activation, we used a late-stage autophagy inhibitor, chloroquine (CQ), a lysosomotropic agent that inhibits binding between lysosomes and autophagosomes [30], and measured autophagic activities through immunostaining and immunoblotting assays. CQ treatment of cells either inhibits the acidification of cell lysosomes or prevents autophagosome structures from binding with lysosomes [31]. While OxyR treatment caused significantly higher numbers of LC3 puncta, CQ significantly inhibited the binding of autophagosome and lysosome; significantly more LC3 puncta were therefore formed in astrocytes. In cells treated with both OxyR and CQ, LC3 puncta formation was significantly more pronounced in astrocytes (Figure 1A,B), indicating that the autophagic flux was augmented by OxyR. To confirm these effects, we also performed immunoblot analysis in OxyR-treated astrocytes. The immunoblotting results indicated that co-treatment with OxyR and CQ additionally upregulated LC3-II expression in cortical astrocytes (Figure 1C,D), suggesting that OxyR exhibited autophagic activities. However, to confirm autophagy, we performed a separate autophagic flux experiment in rat cortical primary neurons using immunofluorescence and immunoblotting assays. We found that OxyR + CQ co-treated cells formed significantly more LC3 puncta and expressed significantly more LC3-II protein (Figure 1E–H), implying that OxyR increased autophagic flux in rat cortical neurons. An immunoblotting-based analysis of p62 showed that OxyR treatment caused significantly lower p62 expression in both astrocytes and neurons. By performing the LC3 turnover assay, we similarly found that autolysosome accumulation was prevented by CQ; as a result, p62 levels were considerably increased after treatment with OxyR, due to the impaired deprivation of p62 (Figure 1C,D,G,H). Therefore, our findings suggest that OxyR stimulates autophagic flux both in cortical astrocytes and neurons.

### 3.2. OxyR Activates the Autophagic Pathway in Mouse Cortical Astrocytes and Rat Cortical Neurons

**Figure 2:**
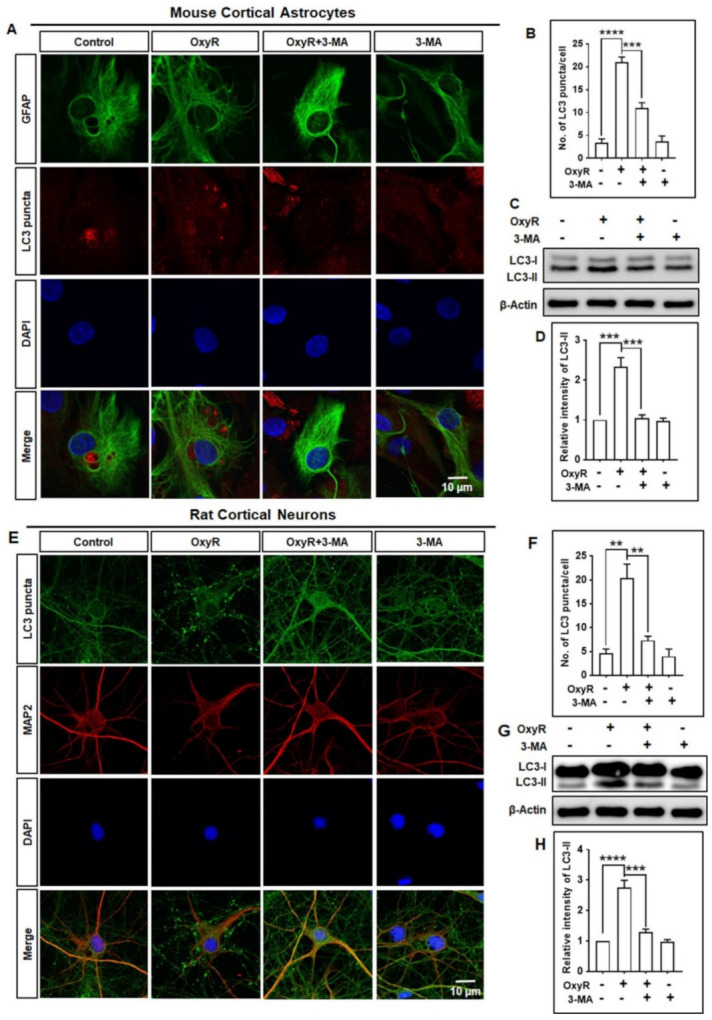
The autophagy inhibitor 3-MA blocked OxyR-mediated autophagy in cortical astrocytes and neurons. (**A**,**E**) Astrocytes and neurons were pretreated with 3-MA (2.5 mM) for 3 h, and LC3 puncta were visualized by immunofluorescence staining with anti-GFAP (green), anti-MAP2 (red), and anti-LC3 (red) antibodies under a confocal microscope. (**B**,**F**) LC3 puncta formation was determined from at least 3 randomly selected independent areas on each slide; the puncta of at least 5 cells were counted. (**C**,**G**) Astrocytes and neurons were pretreated with 3-MA for 3 h; OxyR treatment included incubation with 10 µM OxyR for 24 h. Representative LC3 levels were determined by immunoblotting analysis. (**D**,**H**) Western blot band intensities were measured by using ImageJ software, and each expression was normalized to that of β-actin. Statistical analysis was performed by one-way ANOVA (*n* = 3). Data are presented as mean ± SEM. ** *p* < 0.01, *** *p* < 0.001, **** *p* < 0.0001. OxyR, oxyresveratrol; 3-MA, 3-methyladenine; GFAP, glial fibrillary acidic protein; MAP2, microtubule-associated protein 2; DAPI, the nuclear marker 4′,6-diamidino-2-phenylindole; LC3, microtubule-associated protein light chain 3.

To elucidate the effects of OxyR on autophagy, we evaluated whether the autophagy inhibitor 3-methyladenine (3-MA), a well-recognized class III phosphatidylinositol 3-kinase (PI3K) inhibitor commonly used in autophagy inhibition [32], regulated autophagy in cortical astrocytes. We found that OxyR caused significantly higher LC-3 puncta formation in cortical astrocytes. Particularly, treatment with both 3-MA and OxyR resulted in significantly lower numbers of LC-3 puncta, based on immunofluorescence assays (Figure 2A,B). Additionally, immunoblotting analysis showed that LC3-II protein expression was significantly higher in the presence of OxyR, significantly lower under OxyR + 3-MA co-treatment, and unchanged in the presence of 3-MA (Figure 2C,D). Furthermore, to confirm OxyR-mediated autophagy activity, we performed additional immunofluorescence and immunoblotting analyses using rat cortical primary neurons. These showed that OxyR treatment resulted in significantly more LC-3 puncta counts and higher LC3-II expression, and that 3-MA co-treatment significantly blocked OxyR-induced LC-3 puncta formation and LC3-II expression in rat cortical neurons (Figure 2E–H). Together, these results indicate that OxyR-mediated autophagy is dependent on the class III PI3K pathway in cortical astrocytes and neurons.

### 3.3. OxyR-Mediated Autophagy Is Dependent on the AMPK-mTOR Signaling Pathway in Mouse Cortical Astrocytes

**Figure 3:**
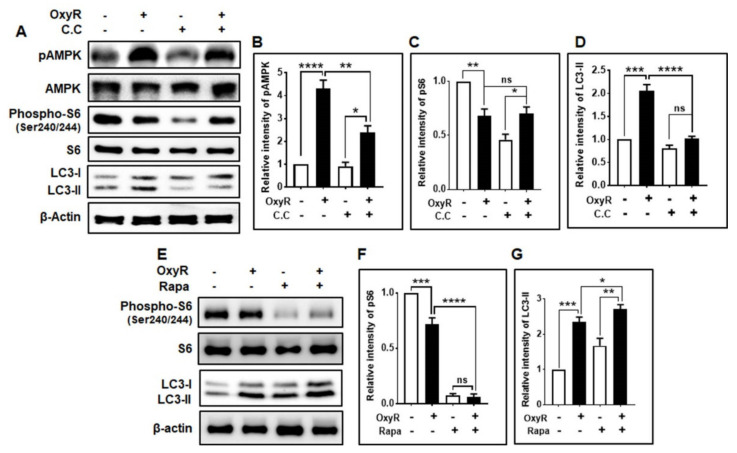
OxyR-activated autophagy via the AMPK)-mTOR pathway in cortical astrocytes. (**A**) Astrocytes were pretreated with compound C (10 µM) for 1 h in the presence or absence of 10 µM OxyR for a further 24 h. pAMPK, AMPK, phospho-S6 ribosomal protein (Ser240/244), S6 ribosomal protein, and LC3 expression levels were determined by performing immunoblotting. (**B**–**D**) Densitometry analyses of the represented proteins were performed using ImageJ, and individual expression levels were normalized to those of β-actin. Statistical analysis was accomplished by one-way ANOVA. (**E**) Cells were pretreated with rapamycin for 30 min before OxyR (10 µM) treatment for 24 h. Phospho-S6, S6, and LC3 levels were measured by performing immunoblotting. (**F**,**G**) All data were derived from one-way ANOVA. Data are presented as mean ± SEM. * *p* < 0.05, ** *p* < 0.01, *** *p* < 0.001, **** *p* < 0.0001, ns: non-significant. OxyR, oxyresveratrol; C.C, compound C (AMPK inhibitor); AMPK, AMP-activated protein kinase; pAMPK, phosphorylated AMPK; LC3, microtubule-associated protein light chain 3; Rapa, rapamycin; S6, S6 ribosomal protein; pS6, phosphorylated S6.

To understand the molecular mechanisms involved in autophagy activation induced by OxyR treatment, we assessed whether this polyphenolic compound regulated the expression of the autophagy initiation proteins AMPK and mTOR in astrocytes. AMPK has been found to initiate autophagy and maintain cellular energy and homeostasis [33] in response to numerous cellular stresses [34]. In general, AMPK activates autophagy via inhibition of mTOR [35], but the mechanism by which AMPK regulates autophagy remains elusive. We found that OxyR treatment caused significantly higher AMPK phosphorylation by upregulating LC3-II expression, as determined by immunoblotting analysis (Figure 3A,B). We also checked mTOR downstream phospho-S6 ribosomal protein (Ser240/244) (Figure 3A,C). However, co-treatment with OxyR and compound C [36], an AMPK inhibitor, and OxyR caused significantly lower AMPK phosphorylation, with non-significantly decreased phospho-S6 (Figure 3A,C), and LC3-II expression (Figure 3A,D), indicating that AMPK controlled the OxyR-mediated autophagic pathway in cortical astrocytes. Additionally, we investigated whether autophagy was controlled by the mTOR pathway in cortical astrocytes. We found that the expression level of mTOR downstream phospho-S6 ribosomal protein (Ser240/244) was significantly lower in the OxyR-treated cells via higher expression of the autophagic marker LC3-II (Figure 3E,F). Meanwhile, co-treatment with the mTOR inhibitor rapamycin caused both significantly lower phospho-S6 ribosomal protein levels and upregulated LC3-II expression (Figure 3E,G), suggesting that mTOR was involved in OxyR-induced autophagy. Together, these results indicate that OxyR-mediated autophagy is dependent on the AMPK-mTOR pathway in mouse cortical astrocytes.

### 3.4. OxyR Activates the Autophagy Initiation Protein ULK1 in Mouse Cortical Astrocytes

**Figure 4:**
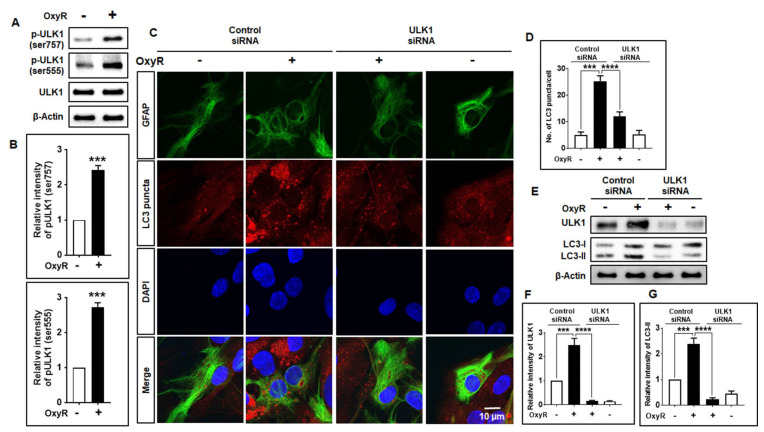
OxyR-mediated autophagy initiated via the ULK1-dependent pathway in cortical astrocytes. (**A**) Astrocytes were cultured in the presence or absence of OxyR (10 µM) for 24 h. ULK1, pULK1 (ser757), and pULK1 (ser555) expression levels were examined by Western blotting. (**B**) Densitometry analysis was performed by using ImageJ software. Statistical analysis was accomplished by conducting one-way ANOVA. (**C**) Control siRNA and ULK1 siRNA were transfected for 48 h and incubated with 10 µM of OxyR for 24 h. Astrocytes were fixed and stained with Alexa Fluor-conjugated anti-GFAP mouse mAb (green) and Alexa Fluor-conjugated anti-LC3 antibody (red). Images were acquired using a confocal microscope. (**D**) LC3 puncta were counted in three independent, randomly selected areas, and at least 5 cells were counted. (**E**) Representative ULK1 and LC3 expression levels were determined by Western blotting. (**F**,**G**) Densitometry and statistical analysis were performed using ImageJ and one-way ANOVA, respectively. Data are presented as mean ± SEM. *** *p* < 0.001, **** *p* < 0.0001. OxyR, oxyresveratrol; ULK1, unc-51-like autophagy activating kinase 1; p-ULK1, phosphorylated ULK1; siRNA, small interfering RNA; GFAP, glial fibrillary acidic protein; LC3, microtubule-associated protein light chain 3; DAPI, the nuclear marker 4′,6-diamidino-2-phenylindole.

To determine whether the autophagy initiation protein complex ULK1 was involved in OxyR-induced autophagy in astrocytes, we determined the effects of OxyR in the presence of ULK1 siRNAs. We found that OxyR caused significantly higher phosphorylation of ULK1 (Ser757) and ULK1 (Ser555) in astrocytes (Figure 4A,B). Further UKL1 siRNA experiments were conducted to determine whether OxyR-mediated autophagy induction was dependent on the ULK1 pathway. Here, using immunocytochemical assays, we observed that under the treatment with control siRNAs, OxyR caused the formation of more LC3 puncta. However, ULK1 siRNA alone caused significantly lower OxyR-induced LC3 puncta formation in cortical astrocytes (Figure 4C,D). Moreover, immunoblotting analysis indicated that co-treatment of cells with OxyR and ULK1 siRNA resulted in significantly lower ULK1 and LC3-II expression levels in cortical astrocytes (Figure 4E–G). Together, these results indicate that ULK1 is an essential component of OxyR-mediated autophagy regulation in mouse cortical astrocytes.

### 3.5. OxyR Activates Lysosomal Protein LAMP1 in Mouse Cortical Astrocytes

**Figure 5:**
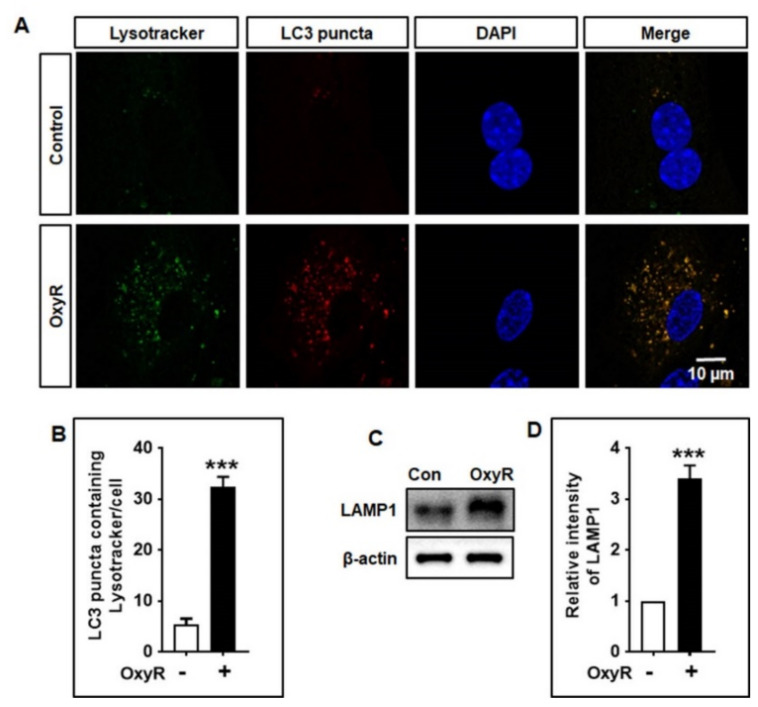
OxyR promoted autophagosome–lysosome fusion in astrocytes. (**A**) After 24 h of OxyR treatment, LysoTracker Green-HCK-123 was added and the culture was maintained at 37 °C for 2 h before fixation. (**B**) From each slide, 3 random areas were selected and the number of LC3 puncta containing LysoTracker was counted in each and analyzed using one-way ANOVA. (**C**) LAMP1 expression levels were quantified using Western blotting. (**D**) Statistical significance was revealed by one-way ANOVA. Data are presented as mean ± SEM. *** *p* < 0.001. OxyR, oxyresveratrol; LC3, microtubule-associated protein light chain 3; DAPI, the nuclear marker 4′,6-diamidino-2-phenylindole; LAMP1, lysosomal-associated membrane protein 1.

To complete the formation of the autophagolysosome, binding of the lysosome membrane proteins with autophagosomes and lysosomes is necessary [37]. Autophagolysosomes are damaged when lysosomal protein levels are spontaneously reduced or are selectively reduced during the fusion of autophagosome and lysosome [38]. To study whether lysosomes were associated with the occurrence of OxyR-mediated autophagy, we detected lysosomes using a green fluorescent lysosomal detection marker, LysoTracker, which labels and tracks the acidic organelles of living cells. We found that OxyR-treated cells exhibited significantly more LC3 puncta containing LysoTracker in cortical astrocytes using double immunofluorescence staining (Figure 5A,B). Additionally, to confirm the involvement of lysosomes which fused with autophagosomes, we examined lysosomal-associated membrane protein 1 (LAMP1) using immunoblotting analyses. Our results suggested that OxyR treatment significantly upregulated LAMP1 protein expression in cortical astrocytes (Figure 5C,D). Thus, this study indicates that OxyR-mediated autophagy stimulates lysosomal protein in mouse cortical astrocytes.

### 3.6. OxyR Decreases Glucocorticoid-Induced APP Expression via the Autophagy Pathway in Mouse Cortical Astrocytes

**Figure 6:**
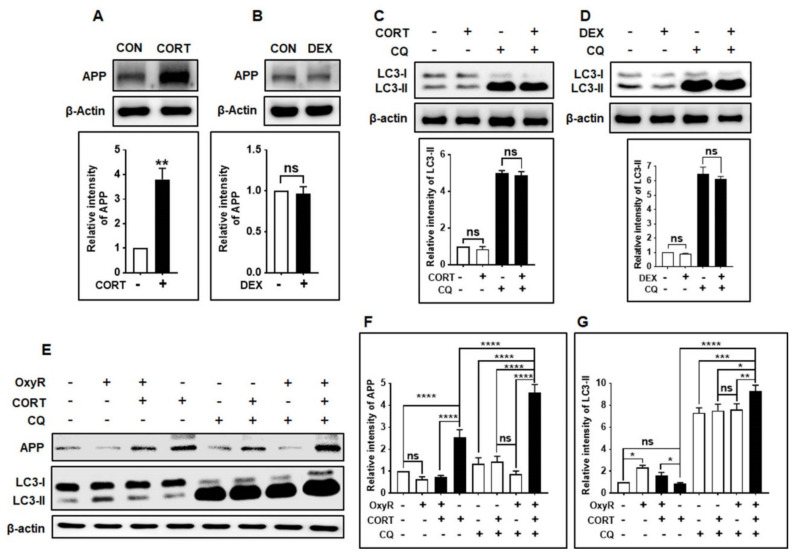
OxyR reduced APP production through the autophagy pathway. (**A**,**B**) Astrocytes were treated with 10 µM of CORT and DEX for 48 h. APP expression was determined by Western blotting. The intensity of the APP signal was measured by using ImageJ. (**C**,**D**) After conducting CORT and DEX treatments for 48 h, 10 µM CQ was added 2 h before harvest. The presence of LC3 was determined by Western blotting. (*n* = 3). (**E**) Astrocytes were treated with CORT for 48 h, and then with 10 µM OxyR for 24 h. CQ treatment (10 µM) was performed 2 h before cell harvest. The presence of APP and LC3 was determined by Western blotting. (**F**,**G**) Statistical analysis was performed. Data are presented as mean ± SEM. * *p* < 0.05, ** *p* < 0.01, *** *p* < 0.001, **** *p* < 0.0001, ns: non-significant. CON, control; CORT, corticosterone; DEX, dexamethasone; APP, amyloid precursor protein; CQ, chloroquine; LC3, microtubule-associated protein light chain 3.

Finally, we examined whether OxyR-induced autophagy was capable of reducing APP production in the mouse cortical astrocyte model. Previous studies have suggested that astrocytes can directly contribute to amyloid plaque production in the adult mouse astrocyte model [39,40]. Wang et al. reported that the glucocorticoid corticosterone (CORT) and synthetic glucocorticoid dexamethasone (DEX) [41] induced APP and BACE1 production in adult mouse astrocytes [17]. Therefore, we determined whether CORT or DEX affected APP production in our cultured mouse astrocyte model, using immunoblotting assays. Firstly, we checked and compared the APP-producing effects of CORT and DEX in astrocytes. We found that CORT induced significantly more APP production than DEX (Figure 6A,B). Furthermore, neither CORT nor DEX alone could increase the autophagic activity (Figure 6C,D) in mouse cortical astrocytes. OxyR treatment alone did not cause significantly lower APP production, while CORT + OxyR co-treatment caused significantly lower APP production (Figure 6E,F), indicating that OxyR decreased CORT-induced APP production. Furthermore, LC3 production was significantly increased in CORT + OxyR co-treated cells, suggesting that OxyR restrained autophagy in astrocytes (Figure 6E,G). To determine whether decreased APP production and restrained LC3 expression were directly associated with autophagy signaling, we performed autophagic flux experiments with the late-stage autophagy inhibitor CQ after exposing astrocytes to CORT. As expected, OxyR + CORT + CQ co-treated cells showed significantly more APP expression than CORT + OxyR co-treated cells, suggesting that autophagy inhibition restrained APP production (Figure 6E,F). However, OxyR caused additional accumulated LC3 expression compared to that in CORT + CQ co-treated cells, indicating that the OxyR-mediated APP reduction was dependent on the autophagy pathway. Taken together, these results indicate that CORT-induced APP production is diminished by OxyR treatment in mouse cortical astrocytes.

## 4. Discussion

The present study provides evidence that APP-induced stress in cultured mouse cortical astrocytes can be prevented by OxyR via the autophagy pathway. Previously, it has been demonstrated that OxyR conferred protection to rat cortical neuronal cells against Aβ-induced apoptotic cell death [6]. In the present study, we found that OxyR could induce the AMPK/ULK1/mTOR dependent autophagy pathway to attenuate APP production in a mouse cortical astrocyte model. However, the exact molecular mechanism by which OxyR causes CORT-induced APP reduction through the autophagy pathway remains elusive and this aspect warrants further investigation.

In autophagy studies, it is important to distinguish whether autophagosome accumulation occurs due to a blockage of the late-stage autophagy inhibitor CQ, termed “autophagic flux” analysis [42]. Generally, immunoblotting analysis of endogenous LC3-II expression is considered one of the key assays for quantifying autophagic flux [42]. Autophagosome–lysosome fusion inhibitors such as E-64d/peps A, bafilomycin A1, or chloroquine, have been found to block this conjugation, which might enhance LC3-II expression. We found that co-treatment with OxyR and CQ enhanced LC3-II puncta formation and LC3-II protein expression (Figure 1). Particularly, we found that the difference between LC3-II levels in the presence and absence of CQ was greater under conditions of OxyR co-treatment (Figure 1), indicating that OxyR improved autophagic flux in cortical astrocytes. This is consistent with our previous study, in which gintonin improved fluorescently tagged LC3-II puncta formation in mouse cortical astrocytes treated with a late-stage autophagy inhibitor [25]. Therefore, the present results suggest that the natural antioxidant, OxyR, can increase autophagic flux in cortical astrocytes.

The autophagy-initiating proteins AMPK and ULK1 are necessary for the regulation of autophagy. AMPK is generally activated under starvation conditions and inhibits mTOR expression, leading to ULK1-AMPK interaction [43]. It has been proposed that this AMPK–ULK1 interaction is critical for the regulation and induction of autophagy. Furthermore, AMPK phosphorylates ULK1 and activates autophagy initiation [44]. We observed that OxyR treatment activated AMPK and stimulated autophagy, while inhibition of AMPK by compound C did not affect OxyR-mediated autophagy induction (Figure 3), indicating that AMPK played an important role in autophagy initiation and regulation in cortical astrocytes. However, mTOR plays an essential role in AD pathogenesis, which contributes to cognitive impairment as well as brain dysfunction [45], although mTOR inhibition protects the pathogenesis of AD [20]. OxyR has previously been reported to activate autophagy in neuroblastoma cells via an mTOR-dependent pathway [4]. In the present study, we showed that co-treatment with rapamycin and OxyR resulted in considerably lower mTOR levels and more autophagy (Figure 3). Thus, our findings demonstrate that OxyR-induced autophagy is primarily dependent on the mTOR pathway in cortical astrocytes. This is consistent with our previous results, which have indicated that gintonin-mediated autophagy is dependent on autophagy signaling in mouse cortical astrocytes [25].

It has been found that the ULK1 complex is essential during the early-stage initiation of autophagy [46]. Additionally, accumulating evidence suggests that a relationship exists between ULK1 and mTOR in mammalian cells [43]. ULK1 may be phosphorylated by mTOR or AMPK, which initiates autophagy [43,47,48]. Moreover, under high-nutrient conditions, mTOR inhibits ULK1 by phosphorylating its Ser757 position, thereby disrupting the interaction of ULK1 and AMPK that is important for ULK1-mediated autophagy initiation [43]. Our investigation found that OxyR treatment augmented ULK1 phosphorylation and regulated autophagy induction. Particularly, application of ULK1 siRNAs resulted in significantly fewer LC3 puncta counts and lower LC3-II protein expression in cortical astrocytes (Figure 4), thereby indicating the essential role of ULK1 in regulating OxyR-mediated autophagy in this model. It has previously been stated that the natural compound 18α-glycyrrhetinic acid and OxyR-mediated autophagy display accumulation of Atg5, Atg7, and Beclin-1 in neuroblastoma cells [4,49]. Furthermore, autophagosome–lysosome fusion, via association with lysosomal proteins, is necessary to complete the autophagic process. Lysosomal-associated membrane protein 1 (LAMP1) is found in the lysosomal membrane, playing an important role in binding with autophagosomes [50] and activating autophagic degradation. Interestingly, we found that OxyR activated LAMP1 protein expression, based on LysoTracker staining results in astrocytes (Figure 5). Therefore, the present findings suggest that OxyR is important for the regulation of autophagy in the lysosome-mediated fusion of mouse cortical astrocytes.

There are no universally recognized pharmacological agents available that may result in direct inhibition of APP and Aβ production in AD pathogenesis, and it is essential to find a pharmacological mediator to delay AD progression. OxyR has been found to restrict the occurrence of oxidative stress and inflammation in the rat model of spinal cord injury [51]. Previously, it has been reported that CORT facilitated astrocytic Aβ peptide accumulation by enhancing APP and BACE1 expression [17]. In the present study, we found that CORT-induced APP production was attenuated by OxyR via the AMPK/ULK1/mTOR-mediated autophagy pathway in adult mouse cortical astrocytes. Herein, we provide the first evidence that OxyR alleviates CORT-induced APP production via activation of the autophagy pathway (Figure 6). However, our previous study showed that OxyR-induced autophagy in human neuroblastoma cells independently of apoptosis [4].

## 5. Conclusions

**Figure 7:**
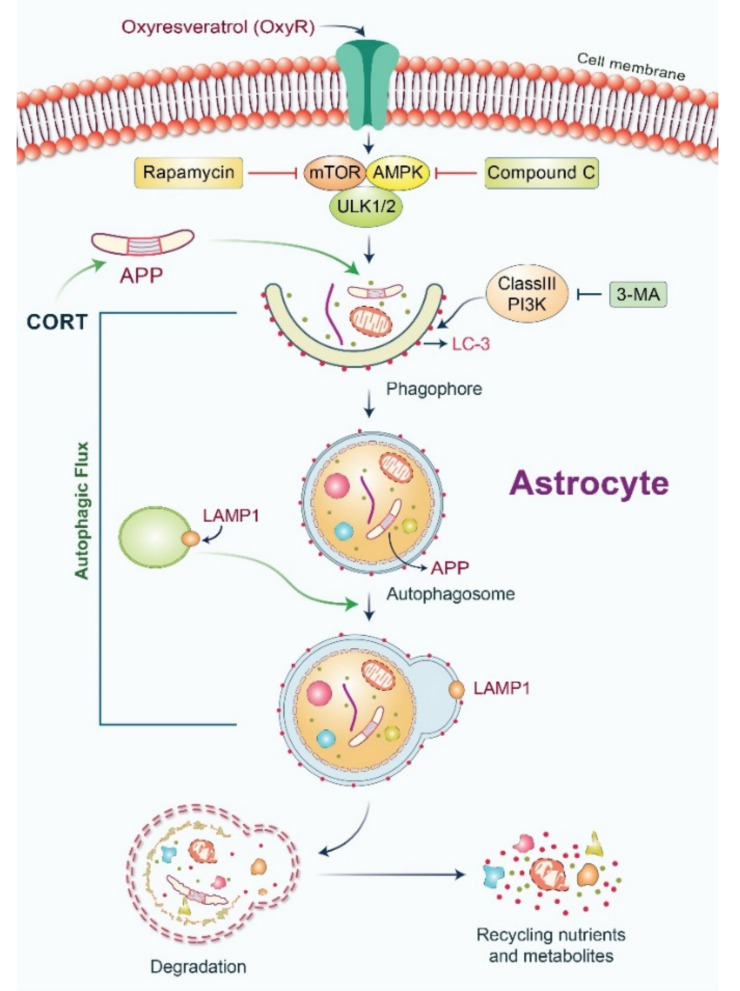
Model of OxyR-mediated APP reduction and autophagy induction in cortical astrocytes. OxyR initiates autophagy via stimulation of the AMPK/ULK1/mTOR pathway to activate phagophore formation and subsequently autophagosome maturation. CORT-initiated APP expression is followed by APP engulfment by autophagosomes. The lysosomal protein LAMP1 supports the binding of lysosomes with autophagosomes. Finally, APP is degraded by the autophagosome-lysosomal pathway, and the released nutrients and metabolites are recycled. AMPK, AMP-activated protein kinase; mTOR, mammalian target of rapamycin; ULK1, Unc-51-like autophagy activating kinase 1; APP, amyloid precursor protein; ClassIII PI3K, class III PI3-kinase; 3-MA, 3-methyladenine; LAMP1, lysosomal-associated membrane protein 1.

In conclusion, it can be inferred that OxyR-mediated autophagy pathways are regulated via AMPK/ULK1/mTOR-dependent signaling in mammalian brain cells. Our proposed model for CORT-induced APP production, as well as AMPK/ULK1/mTOR-mediated autophagy regulation by OxyR, is presented in Figure 7. Although further investigation is warranted to elucidate the detailed molecular mechanism underlying the phosphorylation of ULK1 with its AMPK interaction and mTOR regulation to modulate OxyR-induced autophagy, our results provide evidence for an association between OxyR and CORT-induced APP reduction in astrocytes via the autophagy pathway.

## Data Availability

The data presented in this study are available on request from the corresponding author. The data are not publicly available due to Institutional guideline.

## References

[1] Andrabi A.S., Spina M.G., Lorenz P., Ebmeyer U., Wolf G., Horn T.F. (2004). Oxyresveratrol (trans-2,3′,4,5′-tetrahydroxystilbene) is neuroprotective and inhibits the apoptotic cell death in transient cerebral ischemia. Brain Res..

[2] Qiu F., Komatsu K., Kawasaki K., Saito K., Yao X., Kano Y. (1996). A Novel Stilbene Glucoside, Oxyresveratrol 3′-*O*-β-Glucopyranoside, from the Root Bark ofMorus alba. Planta Med..

[3] Lorenz P., Roychowdhury S., Engelmann M., Wolf G., Horn T.F. (2003). Oxyresveratrol and resveratrol are potent antioxidants and free radical scavengers: Effect on nitrosative and oxidative stress derived from microglial cells. Nitric Oxide.

[4] Rahman A., Bishayee K., Sadra A., Huh S.-O. (2017). Oxyresveratrol activates parallel apoptotic and autophagic cell death pathways in neuroblastoma cells. Biochim. Biophys. Acta (BBA) Gen. Subj..

[5] Breuer C., Wolf G., Andrabi S.A., Lorenz P., Horn T.F. (2006). Blood–brain barrier permeability to the neuroprotectant oxyresveratrol. Neurosci. Lett..

[6] Ban J.Y., Jeon S.-Y., Nguyen T.T.H., Bae K., Song K.-S., Seonga Y.H. (2006). Neuroprotective Effect of Oxyresveratrol from Smilacis Chinae Rhizome on Amyloid. BETA. Protein (25-35)-Induced Neurotoxicity in Cultured Rat Cortical Neurons. Biol. Pharm. Bull..

[7] Jeon S.-Y., Kwon S.-H., Seong Y.-H., Bae K., Hur J.-M., Lee Y.-Y., Suh D.-Y., Song K.-S. (2007). β-secretase (BACE1)-inhibiting stilbenoids from Smilax Rhizoma. Phytomedicine.

[8] Rahman A., Rahman S., Uddin J., Mamum-Or-Rashid A.N.M., Pang M.-G., Rhim H. (2020). Emerging risk of environmental factors: Insight mechanisms of Alzheimer’s diseases. Environ. Sci. Pollut. Res..

[9] Rodríguez J.J., Olabarria M., Chvatal A., Verkhratsky A., Rodr J.J. (2008). Astroglia in dementia and Alzheimer’s disease. Cell Death Differ..

[10] Söllvander S., Nikitidou E., Brolin R., Söderberg L., Sehlin D., Lannfelt L., Erlandsson A. (2016). Accumulation of amyloid-β by astrocytes result in enlarged endosomes and microvesicle-induced apoptosis of neurons. Mol. Neurodegener..

[11] Rahman A., Hwang H., Cho Y., Rhim H. (2019). Modulation of O-GlcNAcylation Regulates Autophagy in Cortical Astrocytes. Oxidative Med. Cell. Longev..

[12] Frost G.R., Li Y.-M. (2017). The role of astrocytes in amyloid production and Alzheimer’s disease. Open Biol..

[13] Lööv C., Hillered L., Ebendal T., Erlandsson A. (2012). Engulfing Astrocytes Protect Neurons from Contact-Induced Apoptosis following Injury. PLoS ONE.

[14] Chung W.-S., Clarke L.E., Wang G.X., Stafford B.K., Sher A., Chakraborty C., Joung J., Foo L.C., Thompson A., Chen C. (2013). Astrocytes mediate synapse elimination through MEGF10 and MERTK pathways. Nat. Cell Biol..

[15] Zhao J., Connor T.O., Vassar R. (2011). The contribution of activated astrocytes to Aβ production: Implications for Alzheimer’s disease pathogenesis. J. Neuroinflamm..

[16] Guo T., Zhang D., Zeng Y., Huang T.Y., Xu H., Zhao Y. (2020). Molecular and cellular mechanisms underlying the pathogenesis of Alzheimer’s disease. Mol. Neurodegener..

[17] Wang Y., Li M., Tang J., Song M., Xu X., Xiong J., Li J., Bai Y. (2011). Glucocorticoids Facilitate Astrocytic Amyloid-β Peptide Deposition by Increasing the Expression of APP and BACE1 and Decreasing the Expression of Amyloid-β-Degrading Proteases. Endocrinology.

[18] Rahman M., Hossain M., Biswas P., Islam R., Uddin M.J., Rahman M.D., Rhim H. (2020). Molecular Insights into the Multifunctional Role of Natural Compounds: Autophagy Modulation and Cancer Prevention. Biomedicines.

[19] Tong X., Ganta R.R., Liu Z. (2020). AMP-activated protein kinase (AMPK) regulates autophagy, inflammation and immunity and contributes to osteoclast differentiation and functionabs. Biol. Cell.

[20] Uddin S., Rahman A., Kabir T., Behl T., Mathew B., Perveen A., Barreto G.E., Bin-Jumah M.N., Abdel-Daim M.M., Ashraf G.M. (2020). Multifarious roles of mTOR signaling in cognitive aging and cerebrovascular dysfunction of Alzheimer’s disease. IUBMB Life.

[21] Yan Q., Han C., Wang G., Waddington J.L., Zheng L., Zhen X. (2017). Activation of AMPK/mTORC1-Mediated Autophagy by Metformin Reverses Clk1 Deficiency-Sensitized Dopaminergic Neuronal Death. Mol. Pharmacol..

[22] Turco E., Fracchiolla D., Martens S. (2020). Recruitment and Activation of the ULK1/Atg1 Kinase Complex in Selective Autophagy. J. Mol. Biol..

[23] Singha B., Laski J., Valdés Y.R., Liu E., DiMattia G.E., Shepherd T.G. (2020). Inhibiting ULK1 kinase decreases autophagy and cell viability in high-grade serous ovarian cancer spheroids. Am. J. Cancer Res..

[24] Rahman A., Saha S.K., Rahman S., Uddin J., Uddin S., Pang M.-G., Rhim H., Cho S.-G. (2020). Molecular Insights Into Therapeutic Potential of Autophagy Modulation by Natural Products for Cancer Stem Cells. Front. Cell Dev. Biol..

[25] Rahman A., Hwang H., Nah S.-Y., Rhim H. (2020). Gintonin stimulates autophagic flux in primary cortical astrocytes. J. Ginseng Res..

[26] Rahman A., Cho Y., Hwang H., Rhim H. (2020). Pharmacological Inhibition of O-GlcNAc Transferase Promotes mTOR-Dependent Autophagy in Rat Cortical Neurons. Brain Sci..

[27] Honda S., Arakawa S., Yamaguchi H., Torii S., Sakurai H.T., Tsujioka M., Murohashi M., Shimizu S. (2020). Association Between Atg5-independent Alternative Autophagy and Neurodegenerative Diseases. J. Mol. Biol..

[28] Fujikake N., Shin M., Shimizu S. (2018). Association Between Autophagy and Neurodegenerative Diseases. Front. Neurosci..

[29] Cho Y., Hwang H., Rahman A., Chung C., Rhim H. (2020). Elevated O-GlcNAcylation induces an antidepressant-like phenotype and decreased inhibitory transmission in medial prefrontal cortex. Sci. Rep..

[30] Park D., Lee Y. (2014). Biphasic Activity of Chloroquine in Human Colorectal Cancer Cells. Dev. Reprod..

[31] Mauthe M., Orhon I., Rocchi C., Zhou X., Luhr M., Hijlkema K.-J., Coppes R.P., Engedal N., Mari M., Reggiori F. (2018). Chloroquine inhibits autophagic flux by decreasing autophagosome-lysosome fusion. Autophagy.

[32] Heckmann B.L., Yang X., Zhang X., Liu J. (2012). The autophagic inhibitor 3-methyladenine potently stimulates PKA-dependent lipolysis in adipocytes. Br. J. Pharmacol..

[33] Hardie D.G. (2007). AMP-activated/SNF1 protein kinases: Conserved guardians of cellular energy. Nat. Rev. Mol. Cell Biol..

[34] Meley D., Bauvy C., Houben-Weerts J.H., Dubbelhuis P.F., Helmond M.T., Codogno P., Meijer A.J. (2006). AMP-activated Protein Kinase and the Regulation of Autophagic Proteolysis. J. Biol. Chem..

[35] Alers S., Löffler A.S., Wesselborg S., Stork B. (2011). Role of AMPK-mTOR-Ulk1/2 in the Regulation of Autophagy: Cross Talk, Shortcuts, and Feedbacks. Mol. Cell. Biol..

[36] Liu X., Chhipa R.R., Nakano I., Dasgupta B. (2014). The AMPK Inhibitor Compound C Is a Potent AMPK-Independent Antiglioma Agent. Mol. Cancer Ther..

[37] Yim W.W.-Y., Mizushima N. (2020). Lysosome biology in autophagy. Cell Discov..

[38] Ma X., Godar R.J., Liu H., Diwan A. (2012). Enhancing lysosome biogenesis attenuates BNIP3-induced cardiomyocyte death. Autophagy.

[39] Blasko I., Veerhuis R., Stampfer-Kountchev M., Saurwein-Teissl M., Eikelenboom P., Grubeck-Loebenstein B. (2000). Costimulatory Effects of Interferon-γ and Interleukin-1β or Tumor Necrosis Factor α on the Synthesis of Aβ1-40 and Aβ1-42 by Human Astrocytes. Neurobiol. Dis..

[40] Lesné S., Docagne F., Gabriel C., Liot G., Lahiri D.K., Buée L., Plawinski L., Delacourte A., MacKenzie E.T., Buisson A. (2003). Transforming Growth Factor-β1 Potentiates Amyloid-β Generation in Astrocytes and in Transgenic Mice. J. Biol. Chem..

[41] Green K.N., Billings L.M., Roozendaal B., McGaugh J.L., LaFerla F.M. (2006). Glucocorticoids Increase Amyloid-beta and Tau Pathology in a Mouse Model of Alzheimer’s Disease. J. Neurosci..

[42] Mizushima N., Yoshimori T., Ohsumi Y. (2011). The Role of Atg Proteins in Autophagosome Formation. Annu. Rev. Cell Dev. Biol..

[43] Kim J., Kundu M., Viollet B., Guan K.-L. (2011). AMPK and mTOR regulate autophagy through direct phosphorylation of Ulk1. Nat. Cell Biol..

[44] Mao K., Klionsky D.J. (2011). AMPK Activates Autophagy by Phosphorylating ULK1. Circ. Res..

[45] Oddo S. (2012). The role of mTOR signaling in Alzheimer disease. Front. Biosci..

[46] Wang C., Wang H., Zhang D., Luo W., Liu R., Xu D., Diao L., Liao L., Liu Z. (2018). Phosphorylation of ULK1 affects autophagosome fusion and links chaperone-mediated autophagy to macroautophagy. Nat. Commun..

[47] Zachari M., Ganley I.G. (2017). The mammalian ULK1 complex and autophagy initiation. Essays Biochem..

[48] Egan D.F., Kim J., Shaw R.J., Guan K.-L. (2011). The autophagy initiating kinase ULK1 is regulated via opposing phosphorylation by AMPK and mTOR. Autophagy.

[49] Rahman A., Bishayee K., Habib K., Sadra A., Huh S.-O. (2016). 18α-Glycyrrhetinic acid lethality for neuroblastoma cells via de-regulating the Beclin-1/Bcl-2 complex and inducing apoptosis. Biochem. Pharmacol..

[50] Mauvezin C., Neisch A.L., Ayala C.I., Kim J., Beltrame A., Braden C.R., Gardner M.K., Hays T.S., Neufeld T.P. (2016). Coordination of autophagosome–lysosome fusion and transport by a Klp98A–Rab14 complex inDrosophila. J. Cell Sci..

[51] Du H., Ma L., Chen G., Li S. (2017). The effects of oxyresveratrol abrogates inflammation and oxidative stress in rat model of spinal cord injury. Mol. Med. Rep..

